# Reduced brain connectivity along the autism spectrum controlled for familial confounding by co-twin design

**DOI:** 10.1038/s41598-023-39876-y

**Published:** 2023-08-12

**Authors:** Janina Neufeld, Simon Maier, Mirian Revers, Marco Reisert, Ralf Kuja-Halkola, Ludger Tebartz van Elst, Sven Bölte

**Affiliations:** 1 Center of Neurodevelopmental Disorders (KIND), Centre for Psychiatry Research; Department of Women’s and Children’s Health & Stockholm Health Care Services, Karolinska Institutet & Region Stockholm, Stockholm, Sweden; 2 Department for Psychiatry and Psychotherapy, Section for Experimental Neuropsychiatry, Medical Center University of Freiburg, Freiburg, Germany; 3 Department of Stereotactic and Functional Neurosurgery, Medical Center of the University of Freiburg, Medical Faculty, University of Freiburg, Freiburg, Germany; 4 Department of Diagnostic and Interventional Radiology, Medical Physics, Medical Center of the University of Freiburg, Medical Faculty, University of Freiburg, Freiburg, Germany; 5 Department of Medical Epidemiology and Biostatistics, Karolinska Institutet, Stockholm, Sweden; 6 Child and Adolescent Psychiatry, Stockholm Health Care Services, Region Stockholm, Stockholm, Sweden; 7 Curtin Autism Research Group, Curtin School of Allied Health, Curtin University, Perth, WA Australia

**Keywords:** Neuroscience, Psychology, Biomarkers

## Abstract

Previous studies on brain connectivity correlates of autism have often focused on selective connections and yielded inconsistent results. By applying global fiber tracking and utilizing a within-twin pair design, we aimed to contribute to a more unbiased picture of white matter connectivity in association with clinical autism and autistic traits. Eighty-seven twin pairs (n = 174; 55% monozygotic; 24 with clinical autism) underwent diffusion tensor imaging. Linear regressions assessed within-twin pair associations between structural brain connectivity of anatomically defined brain regions and both clinical autism and autistic traits. These were explicitly adjusted for IQ, other neurodevelopmental/psychiatric conditions and multiple testing, and implicitly for biological sex, age, and all genetic and environmental factors shared by twins. Both clinical autism and autistic traits were associated with reductions in structural connectivity. Twins fulfilling diagnostic criteria for clinical autism had decreased brainstem-cuneus connectivity compared to their co-twins without clinical autism. Further, twins with higher autistic traits had decreased connectivity of the left hippocampus with the left fusiform and parahippocampal areas. These associations were also significant in dizygotic twins alone. Reduced brainstem-cuneus connectivity might point towards alterations in low-level visual processing in clinical autism while higher autistic traits seemed to be more associated with reduced connectivity in networks involving the hippocampus and the fusiform gyrus, crucial especially for processing of faces and other (higher order) visual processing. The observed associations were likely influenced by both genes and environment.

## Introduction

Clinical autism, here defined as fulfilling diagnostic criteria for an autism spectrum condition, is characterized by challenges in social and communication functioning along with repetitive behaviors, restricted interests, and alterations in sensory processing^1^. Clinical autism is associated with low employment rates, increased risk of anxiety and depressive disorders, and premature mortality^2^. The heterogeneity of clinical autism and the dimensionality of autism-defining symptoms makes it challenging to establish reliable biomarkers for assessment and intervention purposes. Research indicates that clinical autism is the extreme end of continuously distributed autistic traits^3^. Studying associations with autistic traits in addition to associations with diagnostic markers provides important additional information regarding the brain biomarkers linked to the quantity of autistic features. Hence, we investigated the neural correlates of both, clinical autism and autistic traits in our study.

It is generally assumed that atypical brain development leading to altered brain connectivity underlies the clinical autism phenotypes^4^. The nature of these connectivity alterations is however still largely unknown since neuroimaging findings have hitherto remained inconclusive, with inconsistent findings even across systematic reviews and meta-analyses^5^. This inconsistency can partly be explained by phenotypic variation of clinical autism and methodological heterogeneity^5^. Furthermore, age seems to modulate autism-related white-matter connectivity atypicalities^6^. A developmental model of brain connectivity in clinical autism suggests widespread over-connectivity in early infancy, mirroring findings of early brain overgrowth, followed by altered neurodevelopmental trajectories with regional over- or under-connectivity later in life^7^. In addition, at least some of the brain correlates of clinical autism seem to differ qualitatively between females and males^8^. Finally, environmental factors influencing the likelihood of developing clinical autism, such as parental age or preterm birth^9^, might modulate the brain-structural and functional alterations associated with the condition.

### Structural connectivity correlates of clinical autism and autistic traits

Structural brain connectivity is commonly studied using Diffusion Tensor Imaging (DTI), which takes advantage of the fact that water molecules diffuse along white matter (WM) fibers (i.e. anisotropically). A meta-analysis of voxel-based morphometry across 14 DTI studies, including 297 individuals diagnosed with clinical autism and 302 neurotypical (NT) individuals, revealed decreased fractional anisotropy (FA), indicative of reduced WM integrity, in the left splenium of the corpus callosum and the right cerebral peduncle, possibly reflecting sensorimotor impairments^10^. FA reductions in both genu and splenium of the corpus callosum were also found by another meta-analysis, that in addition found increased mean diffusivity in the posterior thalamic radiation in clinical autism and age effects on callosal alterations across several neurodevelopmental conditions^11^. In contrast, a review of 16 studies applying ‘tract based spatial statistics’ (TBSS), where FA data from DTI images are projected onto a predefined skeleton of prominent fiber tracts, identified more wide-spread reductions in WM connectivity in older children, adolescents and adults diagnosed with clinical autism compared to matched NT controls^6^. The uncinate and arcuate fasciculi, the inferior longitudinal fasciculus, the inferior fronto-occipital fasciculus and the cingulum—tracts crucial for language and face/emotion processing, episodic memory, object recognition and attention control—were particularly affected^6^. In a population-based sample of 604 6–10-year-old children, FA within the left superior longitudinal fasciculus and axial diffusion in the corpus callosum and the corticospinal tract were negatively associated with autistic traits, suggesting that at least some autism-related changes in WM microstructure might show a dose-like effect, increasing with increasing (sub-clinical) levels of autistic traits^12^.

### Utility of twin and sibling designs for assessing autism brain biomarkers

Given the genetic heterogeneity of clinical autism and autistic traits and the difficulties of controlling for all possible confounding variables, twin and sibling studies provide the unique opportunity to implicitly control for genetic and environmental factors. These familial factors might co-occur with clinical autism or correlate with autistic traits without being part of the condition’s phenotype and might have biased the results of previous studies^13^.

Studies including siblings of autistic individuals control implicitly for on average 50% of genetic factors as well as environmental factors shared by siblings, such as socioeconomic background. Such sibling studies indicate that autistic individuals and their siblings without clinical autism show partially overlapping patterns of WM alterations (primarily reductions) compared to NT controls, with siblings typically showing an intermediate phenotype, with fewer or less pronounced alterations^14–16^. One of these studies additionally found increased FA in the inferior longitudinal fasciculus and the corpus callosum in siblings compared to controls, potentially indicating compensatory mechanisms^16^.

Like siblings, dizygotic (DZ) twins share on average half of their genes while monozygotic (MZ) twins share 100% of their genes and all twins share additional environmental factors compared to siblings (for instance influences in utero) and are of the same age. Hence, comparing twins with each other (within-twin pair comparisons) means implicitly controlling for a large number of genetic and environmental factors and age. Further, comparing within-pair associations between MZ and DZ twins allows estimating genetic and environmental influences on brain structure and function^17^. For instance, a meta-analysis of 48 brain imaging studies in NT twins indicated strong genetic impact on cortical morphometric measures and FA of most brain structures, and environmental influence on cortical thickness of the uncus, left parahippocampal gyrus, and insula as well as FA in the callosal splenium^18^. Interestingly, most of the latter structures have also been implicated in clinical autism^6,10,11^.

Only relatively few studies assessed brain structure in twin pairs discordant or concordant for clinical autism, showing, for instance, that autistic twins and their MZ co-twins who did not fulfil diagnostic criteria for clinical autism showed similar reductions in gray matter (GM) and WM volume, WM microstructure and cortical folding^19,20^. This suggests that at least some autism-related brain alterations are likely to be influenced by genetics. Interestingly, the above-mentioned twin and sibling studies relied on group comparisons (autistic individuals, their siblings/twins, and NT control groups) rather than conducting within-pair comparisons, which offer a sensitive approach to identify autism-related alterations beyond familial confounding^21^.

Two recent twin studies applied classical twin modeling to assess the genetic and environmental contributions to the association between clinical autism and brain structure. One study assessing a relatively small and selected sample (33 twin pairs where at least one twin fulfilled diagnostic criteria for clinical autism and 20 NT twin pairs) found evidence for genetic effects on the variability of WM microstructure, where autism-related alterations were detected in the bilateral fornix and left uncinate fasciculus^22^. Another study in a larger twin sample reported that cortical thickness and cerebellar WM volume were more influenced by environmental factors in twins with clinical autism compared to NT twins, whereas the genetic and environmental influences on other brain-structural measures were similar in both twins with and without clinical autism^23^. Previous twin studies from our group revealed intrinsic functional brain connectivity correlates of clinical autism and autistic traits within twin pairs between core hubs of the salience network^21^, and sex-specific within-twin pair associations of brain morphological measures with clinical autism, autistic traits, and specific autism symptom domains^24–26^.

To the best of our knowledge this study is the first to assess within-twin pair associations of WM connectivity with clinical autism and autistic traits using global fiber tracking, reconstructing the entire white-matter connectome without making predetermined anatomical assumptions. While within-twin pair analyses in same-sex twins are perfectly controlled for age and sex, within-pair associations between autism and brain connectivity can be modulated by age^21^ and can differ between males and females^24–26^. Hence, we investigated potential interaction effects between clinical autism/autistic traits and age on brain connectivity, as well as potential sex differences, in follow-up analyses.

## Methods

The Swedish Ethical Review Authority, please see here: https://etikprovningsmyndigheten.se/en/, approved the study protocols. Written informed consent was obtained from all participants and/or their caregivers for both participating in the Roots of Autism and ADHD Twin Study in Sweden (RATSS)^27^ and for publishing the group results based on the thereby acquired data. This study was performed in accordance with the Declaration of Helsinki.

### Participants

A total of 174 individuals (87 twin pairs, 8–36 years) from RATSS were included. In RATSS, twins are predominantly recruited from the population-based Child and Adolescent Twin Study in Sweden (CATSS)^28^, prioritizing pairs who screen positively for autism symptom discordance based on a parent interview. This study included 48 MZ and 39 DZ twin pairs, 24 individuals fulfilled diagnostic criteria for clinical autism, belonging to 16 clinical autism diagnosis discordant (5 MZ and 11 DZ) twin pairs and four concordant twin pairs (3 MZ and 1 DZ). A further 33 individuals without clinical autism had one or more other neurodevelopmental disorder (NDD) (primarily Attention Deficit Hyperactivity Disorder = ADHD, or specific learning disorders) and a further 27 individuals without any NDD fulfilled criteria for one or more other psychiatric diagnosis (mainly affective disorders). The remaining 90 individuals were NT, defined as not fulfilling criteria for any NDD or psychiatric diagnosis. Regarding discordance for autistic traits, 77 twin pairs (39 MZ, 38 DZ) had different total scores on the Social Responsiveness Scale-2 (SRS-2, 29). Of these, 50 twin pairs (23 MZ and 27 DZ) differed by at least 7 points, which is regarded to be above the measurement error for this scale^30^. Sample characteristics are summarized in Table 1. Of originally 420 individuals assessed within RATSS, 230 had to be excluded. The most common exclusion reasons were data quality issues and exclusion because the co-twin was excluded. We also excluded individuals with an IQ below 75, and pairs of opposite sex or where more than one twin pair per family had been assessed (for a detailed description please see the “Exclusion procedure” section in the supplementary text). For a comparison between included and excluded sample, please see Supplementary “Supplementary Results” section.

**Table 1:** Sample characteristics.

	Total sample (n = 174)	Clinical autism (n = 24)	Other diagnoses* (n = 60)	NT (n = 90)
Female/male sex	98/76	11/13	38/22	49/41
MZ/DZ	96/78	11/13	32/28	53/37
Other NDD/psych**	18/51	6/10	33/41	-
Age range years	8–36	11–31	8–36	8–33
Mean age (SD)	19.51 (6.50)	17.33 (5.81)	19.80 (6.85)	19.89 (6.39)
Mean SRS-2 (SD)	32.38 (28.04)	79.62 (25.75)	30.42 (21.33)	21.09 (17.96)
Mean IQ (SD)	103.27 (14.08)	103.38 (18.23)	101.33 (13.08)	104.53 (13.36)

*MZ* monozygotic, *DZ* dizygotic, *Other NDD* neurodevelopmental diagnoses other than autism, *psych* psychiatric diagnoses that are no NDD diagnoses, *SRS-2* social responsiveness scale second version, *IQ* general intellectual ability score from the Wechsler Intelligence scale for adults or children, *NT* neurotypical (defined as not fulfilling diagnostic criteria for any of the assessed neurodevelopmental or other psychiatric diagnoses).

*fulfilling any diagnoses other than clinical autism.

**Other diagnosis can overlap; an individual can contribute to both, other NDD and psychiatric diagnosis count.

### Diagnostic assessment

Twins underwent comprehensive assessment according to the RATSS protocol^27^, including first-choice standardized diagnostic instruments for clinical autism, such as the ‘Autism Diagnostic Interview—Revised’ (ADI-R) and the ‘autism diagnostic observation schedule’ (ADOS or ADOS-2). Other NDD and psychiatric diagnoses were determined based on a multitude of sources, including the ‘Kiddie Schedule for Affective Disorders and Schizophrenia’, the ‘Diagnostic Interview for ADHD in adults’ and the ‘Structured Clinical Interview for DSM-IV’ (SCID, axis I). General intellectual ability was assessed with the Wechsler Intelligence Scale for Children or the Wechsler Adult Intelligence Scale, 4th Editions (WISC-IV/WAIS-IV) and the composite IQ score was calculated based on three verbal comprehension and three perceptual reasoning subtests.

### Assessment of autistic traits

Autistic traits were assessed using the parent-report SRS-2, using total raw scores as recommended for research settings^29^. The SRS-2 consists of 65 items rated by a caregiver on a Likert scale (0–3), focusing on the individual’s behavior during the past six months, with higher scores indicating higher autistic traits. The maximum score is 195 and individuals diagnosed with clinical autism typically score between 60 and 165^30^. The SRS has demonstrated good to excellent psychometric properties for test–retest reliability (0.80–0.97), interrater reliability (0.75–0.95), and satisfactory convergent validity (0.35–0.58) with the ADOS and ADI-R^29–31^. For individuals 8–18 years of age, the school-age child version of the SRS-2 was completed and for individuals of 19 years of age or older the adult version. The child and adult version differ only by a few items where the queried activities and/or social behaviors are not appropriate across ages.

### Image acquisition

An approximately 50-min Magnetic Resonance Imaging (MRI) session in a 3 Tesla MR750 GE scanner included a 5-min T1-weighted Spoiled Gradient Echo anatomical scan (176 slices, TR = 8.2 s, FOV = 240 mm, voxel size = 0.94 × 0.94 × 1.00 mm^3^), and a diffusion imaging sequence with 60 spatial directions and a b-value of 1000 s/mm^2^ (TR = 8.0 s, FOV = 220 mm, Slice Spacing = 0.0 mm, voxel size = 2.29 × 2.29 × 2.3 mm^3^).

### Quality control and processing of the diffusion images

Diffusion images were converted into NRRD (Nearly Raw Raster Data) format and underwent an automatic quality control procedure in DTIPrep (please see https://www.nitrc.org/projects/dtiprep/) in order to identify data quality issues, using DTIPrep’s default protocol settings (tolerance threshold for bad gradients = 0.2, tolerance threshold for missing b-values = 0.005). Data sets with strongly reduced signal to noise ratio were excluded (please see Supplementary text, “Exclusion procedure” section). Data sets from otherwise complete twin pairs that were flagged by DTIPrep for quality issues were visually inspected for artifacts and signal intensity outliers in ExploreDTI4.8.6. Up to three volumes with visible artifacts or sudden drops or peaks in signal intensity were removed from the data (n = 13 participants). If more than three volumes were affected, participants were excluded.

After data pre-processing (motion correction, structural–functional co-registration, eddy-current and motion artifact correction, and spatial normalization), global fiber tracking was performed using the global tracking tool^32^—both within the medical imaging platform NORA (http://www.nora-imaging.com).

### Connectivity assessment

Streamline counts, i.e. the number of reconstructed ‘fibers’, between 56 atlas regions (Supplementary Table S1) of the LBPA40 atlas^33^ were extracted for each individual and normalized (see below). Streamline counts as calculated with the method applied here have previously been demonstrated to be reproducible reliably and can be used as a quantitative measure of structural connectivity^34^.

The streamline counts between all pairs of regions form a symmetric connectivity (or adjacency) matrix, each element of which was normalized by the square-root of the product of row and column sum, i.e. the normalized matrix reads

$$c_{ij}^{\prime } = \frac{{c_{ij} }}{{\sqrt {\left( {s_{i} *s_{j} } \right)} }}$$

where c_ij_ refers to the matrix cells and s_i_ and s_j_ are the row and column sums, respectively. This normalization turns the matrix into the normalized Laplacian of the underlying graph.

### Data reduction

From the connectivity matrix representing the connectivity between the 56 atlas regions (3136 connections), we removed the diagonal (connectivity of each region with itself, 56 connections) and the lower triangle (same connections as upper triangle, 1540 connections). All connections involving the cerebellum were removed due to incomplete coverage of the cerebellum in participants with larger brains (55 connections). In order to further restrict the amount of comparisons to more meaningful connections, we removed all connections where more than 5% of the 90 included NT participants had no fibers at all (340 connections) and the 25% of the remaining connections where the median fiber count in the NT sample was lowest (286 connections), leading to a total of 859 tested connections.

For these, the median raw streamline count across NT participants ranged between 10 and 5145 streamlines.

### Statistical analyses

First, we conducted exploratory analyses on group differences (differences between included and excluded samples as well as between included sub-samples of autistic individuals, individuals with other neurodevelopmental/psychiatric diagnoses and NT individuals) and within-pair differences in IQ. As the main analyses, we performed linear regressions in the generalized estimating equations framework^35^, which do not assume a normal distribution of variables, with streamline counts as dependent variable and clinical autism or autistic traits as independent variables. Using an identity link function and conditioning on a unique twin pair identifier, we conducted within-pair analyses where individuals were compared to their co-twins. Thus, confounding (and mediating) factors that are stable between the twins, i.e. all genetic and environmental factors shared by twins, were adjusted for by design. We fitted models for 859 connections, adjusting for multiple comparisons using false discovery rate (FDR, Benjamini–Hochberg method^36^, as well as for IQ, NDD diagnosis other than clinical autism, and non-NDD psychiatric diagnosis. All 87 twin pairs were included in both the model with clinical autism and the model with autistic traits as main predictor. Twin pairs discordant for the main predictors contributed directly to the estimate (77 pairs were discordant for autistic traits and 16 for clinical autism) while the remaining pairs influenced the standard errors and affected the estimates indirectly if they were discordant for any of the covariates. We then re-ran the models yielding significant within-pair results in the whole cohort also in MZ and DZ sub-samples in order to explore potential genetic effects on the main results. Associations that are only observed in DZ but not MZ twins can be assumed to be influenced by genetics. Standardized estimates were calculated, which can be interpreted as effect size estimates. In a follow-up analysis, the interaction terms between clinical autism or autistic traits and age were added to the models. In order to estimate potential sex effects (more specifically, biological sex assigned at birth based on parent report), we re-ran all models while splitting the sample into male and female twin pairs and comparing the male with the female-specific associations with a Wald-type Chi^2^-test, adjusting the *p*-values for multiple comparisons (859 connections).

In order to complement our within-pair analysis, we also performed linear regressions across the cohort using the same statistical framework, treating twins as individuals but adjusting standard errors for twin clustering, and summarized these secondary results within the supplementary text (“Supplementary results” section).

## Results

### Exploratory results

Exploratory group comparison results comparing the included to the excluded sample and included clinical sub-groups and within-pair differences in IQ are summarized in the “Supplementary results” section (sub-sections 2.1, 2.2. and 2.3).

### Associations of clinical autism and autistic traits with structural brain connectivity

**Figure 1:**
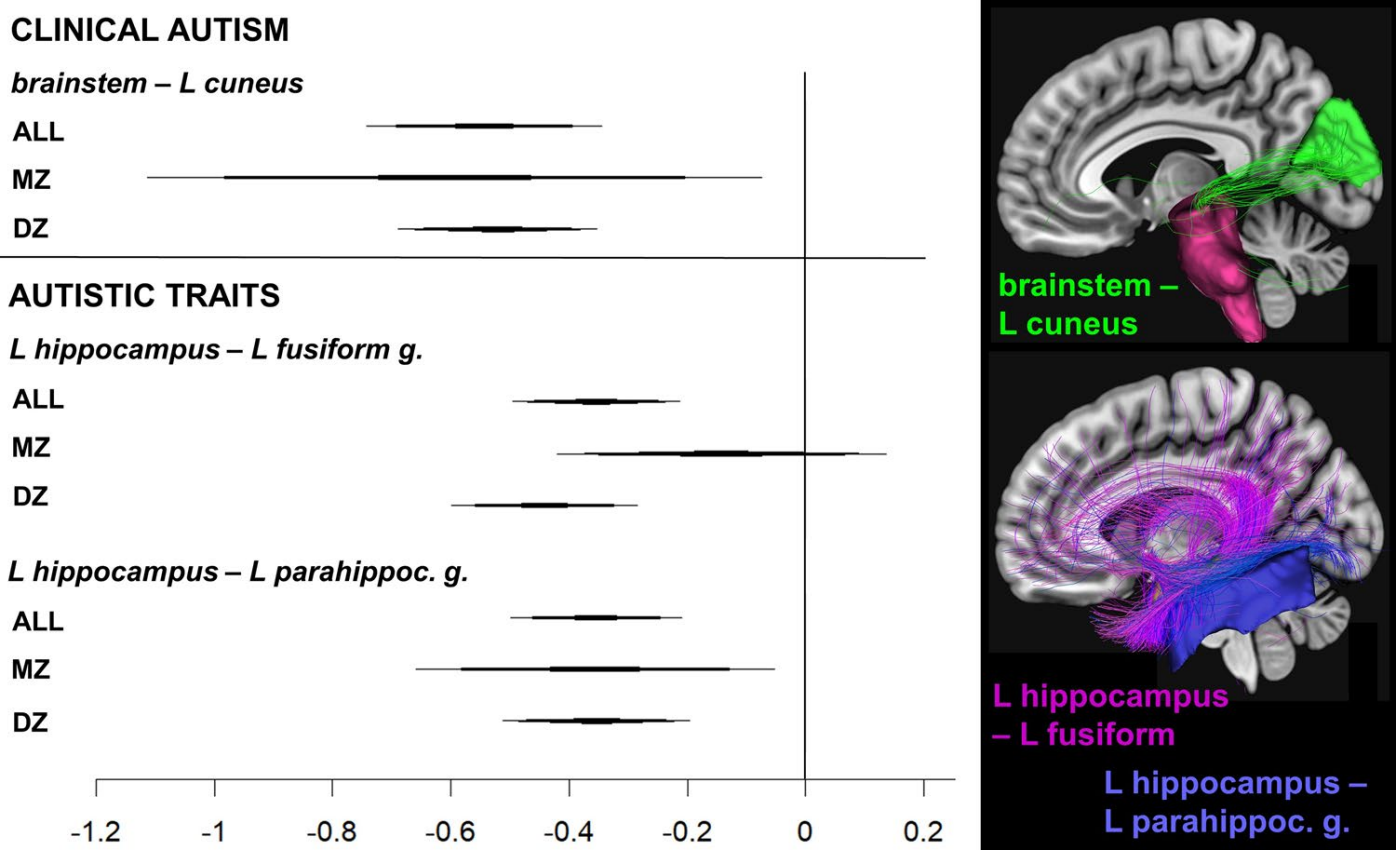
Regression estimates of the main analyses and their confidence intervals. *L *left, *ALL* whole sample, *MZ* within monozygotic twins, *DZ* within dizygotic twins. Note that although none of the associations survived correction within the MZ sub-sample, the confidence intervals cross the zero line only for the association between autistic traits and the L hippocampus—L fusiform gyrus connection. The forest plots were created in RStudio3.5.1, using the package “forestplot”. Examples of the according connections in a single participant (right side) were created using functions within the NORA platform (http://www.nora-imaging.com).

The statistics of within-pair associations with clinical autism or autistic traits surviving the FDR-correction are summarized in Table 2 and visualized in Fig. 1.

**Table 2:** Statistics main analyses.

Sample	Anatomical regions	*b *(95% CI)	SE	p corr	p uncorr
Clinical autism					
ALL	**brainstem—L cuneus**	**− .544 (− .742, − .346)**	**.101**	**7.0 × 10**^**−5**^	**7.8 × 10**^**−8**^
DZ		**− .521 (− .688, − .354)**	**.085**	**9.3 × 10**^**−7**^	**1.0 × 10**^**−9**^
MZ		− .594 (− 1.112, − .075)	.245	1.000	.025
M		− .575 (− .877, − .272)	.155	.180	2.0*10^−4^
F		− .480 (− .738, − .223)	.131	.231	2.6*10^−4^
Autistic traits					
ALL	**L hippocampus—L parahippoc. g**	**− .354 (− .495, − .213)**	**.072**	**8.2 × 10**^**−4**^	**9.2 × 10**^**−7**^
DZ		**− .442 (− .599, − .256)**	**.080**	**2.7 × 10**^**−5**^	**3.0 × 10**^**−8**^
MZ		− .143(− .421, .135)	.142	1.000	.313
M		− .260 (− .435, − .085)	.089	1.000	.004
F		**− .463 (− .637, − .289)**	**.089**	**1.6 × 10**^**−4**^	**1.8 × 10**^**−7**^
ALL	**L hippocampus—L fusiform g**	**− .355 (− .499, − .210)**	**.074**	**.002**	**1.6 × 10**^**−6**^
DZ		**− .354 (− .511, − .196)**	**.080**	**.010**	**1.1 × 10**^**−5**^
MZ		− .356 (− .660, − .053)	.155	1.000	.021
M		− .350 (− .561, − .140)	.107	.990	.001
F		− .359 (− .552, − .166)	.099	.240	2.7 × 10^−4^

*b* regression coefficient, *SE* standard error, *95% CI* 95% confidence interval of the regression coefficient, *p corr.* FDR-corrected p-value; *p uncorr*. uncorrected p-value; *ALL* whole sample, *DZ* within dizygotic twins, MZ within monozygotic twins, M within males, F within females.

Significant associations (corrected *p* < .05) are marked in bold.

In MZ pairs, associations did not survive the FDR-FDRcorrection but pointed into the same direction as in DZ pairs, where they remained significant. When splitting the sample in male and female twin pairs, one of the two associations between connectivity and autistic traits remained significant only in females, but confidence intervals overlapped between females and males.

Clinical autism was significantly associated with decreased structural connectivity between the brainstem and the left cuneus, both within all twin pairs (*corrected p value* < *0.05*) and DZ twin pairs (where 11 were discordant for clinical autism, *corrected p-value* < *.01*). The association was also observable in MZ pairs (where 5 were diagnosis discordant), but did not survive the correction for multiple comparisons. The 95% CIs of the DZ and MZ estimates overlapped. When splitting the sample by biological sex, the association was similar in males and females and did not survive the correction in either group.

There were two significant negative associations between autistic traits and structural connectivity involving the hippocampus (*corrected p-values* < *.001*) that were similar in both the whole sample and the sub-sample of DZ twins (*corrected p-values* < *.01*), whereas the estimate was still negative but not significant in MZ twin pairs. Again, the 95% CIs of the DZ and MZ estimates overlapped. These associations went into the same direction in males and females and the 95% CIs overlapped between sexes. However, the association with connectivity between the left hippocampus and the left parahippocampal gyrus survived the correction only in females, and the association with connectivity between the left hippocampus and the left fusiform gyrus did not survive the correction in either sex sub-group.

The covariates effects are summarized in the “Supplementary results” section (sub-section 2.4), and Supplementary Table S3. Uncorrected Z-values of the associations between clinical autism or autistic traits on connectivity are described in the "Supplementary results” section (sub-section 2.5) and visualized in Supplementary Figures S2 and S3. Effects of the main variables across the cohort (corrected for within-pair relationships) are described in the “Supplementary results” section (sub-section 2.6).

### Modulating effects of age on the associations between clinical autism/autistic traits and structural connectivity

The interaction between age and clinical autism was significant for 15 connections. Among these, however, were none of the connections observed to be linked with clinical autism or autistic traits in the main analysis, but primarily intra-hemispheric fronto-occipital connections (see Supplementary Table S4 and Supplementary Figure S4). All of these interaction effects were negative. Visualizing these effects is challenging, since within-pair associations including several co-variates were modelled. A crude approximation to visualizing the age interaction effects can be found in the supplement (Supplementary Figure S7). No interactions between age and autistic traits survived correction for multiple comparisons. The uncorrected Z-values of the age-interaction effects are visualized in Supplementary Figures S5 and S6.

### Sex differences in the associations between clinical autism/autistic traits and structural connectivity

Across all included connections, no association with clinical autism was significantly different within male compared to female twin pairs. Only one within-pair association with autistic traits was significantly different between males and females, namely the association of autistic traits with WM connectivity linking the brain stem and the right inferior frontal gyrus. In males, the association was negative [b (95% CI) = − .310 (− .549, − .071), SE = .122, uncorrected *p* = .011], in females, the association was positive [b (95% CI) = .450 (.219, .681), SE = .118, uncorrected *p* = 1.3 × 10^–4^]. For an approximation to visualize this sex effect using within-pair differences, please see Supplementary Figure S8.

## Discussion

In this study, we investigated WM connectivity associated with clinical autism and autistic traits, using global fiber tracking and applying a within-twin pair design where familial factors are implicitly controlled for. Both clinical autism and autistic traits were associated with reduced WM connectivity beyond the influence of familial factors. However, different connectivity alterations survived the correction for multiple comparisons for clinical autism based on present diagnostic algorithms compared to autistic traits. The results are discussed in more detail below.

### Clinical autism and structural connectivity

Twins fulfilling diagnostic criteria for clinical autism had reduced WM connectivity between the brainstem and the left cuneus compared to their co-twins without a diagnosis. While several lines of evidence suggest brainstem involvement in clinical autism^37^, direct evidence for brainstem alterations from postmortem histological and in-vivo neuroimaging studies in humans remains limited^37^, likely because the majority of brain imaging studies focused on predetermined cortical regions of interest, often neglecting the brain stem. The brainstem hypothesis of autism suggests that atypical early brainstem development in clinical autism has cascading (bottom-up) effects on cortical development, resulting in alterations in sensory processing that might be causal to other autism core symptoms^37,38^. For instance, the superior colliculus of the brainstem and its interaction with cortical visual regions has been linked to visual exploration during visual search^39^. Such changes in low-level visual processing could have a secondary effect on higher-order visual processing and cognition. The cuneus is an occipital brain region contributing to the dorsal visual stream, involved in form, motion and spatial processing and is a central, integrative hub within a functional visual brain network^40^. This region has previously been implicated in clinical autism in a large-scale study (394 individuals with clinical autism diagnosis and 473 controls). More specifically, lower effective (directed) functional connectivity of the cuneus/precuneus to temporal brain regions involved in face processing was found in relation to both clinical autism and autism symptom severity in the clinical group^41^.

Our finding of reduced connectivity between the brainstem and cuneus in individuals with clinical autism compared to their co-twins might indicate alterations in low-level visual pathway components in clinical autism, which in turn may influence low-level perception and, in consequence, social information processing. These alterations might be considered a marker of the dichotomous presence of clinical autism diagnosis, regardless of symptom severity.

### Autistic traits and structural connectivity

Our results suggest that twins with more pronounced autistic traits tend to have reduced WM connections from the left hippocampus to the left parahippocampal gyrus and to the left fusiform gyrus. The left hippocampus is crucial for (episodic) memory^42^ and is, via the parahippocampal gyrus, connected to the brain’s default mode network^43^, which is believed to be involved in self-referential thinking^44^. Connectivity between the hippocampus and the fusiform gyrus is crucial for facial emotional processing^45^, and reduced connectivity between these regions^46^ and altered microstructure of the hippocampus-fusiform pathway^47^ have been observed previously in individuals diagnosed with clinical autism compared to controls. The human fusiform gyrus contains the fusiform face area (FFA) which is crucial for face perception^48^. Since challenges in facial emotional processing are a core feature of clinical autism, many functional brain imaging studies on clinical autism investigated the FFA during face processing. A meta-analysis of 50 functional MRI studies concluded that the left FFA and the left parahippocampal gyrus are more strongly activated in individuals with clinical autism during social cognition tasks, most of which involving face stimuli^49^. However, the functions of the fusiform gyrus are not restricted to face processing but include for instance also object recognition and space processing^50^. Further, its connectivity to the hippocampus is also relevant for non-social visual processing, such as memory-guided visual exploration in visual search^51^. Therefore, the reduced connectivity between hippocampus and fusiform gyrus in association with increased autistic traits observed in this study might reflect alterations in lower and higher-level visual processing, including, but not restricted to, facial emotional processing. These brain connectivity correlates might be regarded as a marker of quantitative autistic trait severity.

### Genetic vs environmental influences

Comparing associations between MZ and DZ sub-cohorts can allow conclusions regarding the influence of non-shared genetic and environmental factors, since MZ twins share all and DZ twins on average half of their genes. When splitting the sample into sub-samples of 48 MZ and 39 DZ twin pairs, the three negative associations between clinical autism or autistic traits and brain connectivity remained significant within DZ but not MZ twins. Since there were only very few twin pairs discordant for autism diagnosis, the within-pair association with autism diagnosis lacked power especially for the separate zygosity groups. Further, the power for within-MZ effects might have been diminished due to reduced variation in autistic features among MZ twins. However, our previous findings (e.g., reference^21^) demonstrated that associations within MZ samples of similar size are in general detectable. In that context, these associations were most likely predominantly influenced by non-shared environmental factors.

Here, the estimates were quite similar and their 95% CIs overlapped between MZ and DZ sub-cohorts, allowing no firm conclusions with respect to genetic influence on these associations. For the latter, larger MZ and DZ samples will be necessary. Since the associations pointed into the same direction in both MZ and DZ twins but were statistically weaker in MZ twins, we speculate that both genetic and non-shared environmental factors contributed to these within-pair associations.

### Modulating effect of age

Some associations between clinical autism and connectivity between intra-hemispheric connections, involving primarily fronto-occipital connections, were significantly modulated by age (Supplementary Table S4 & Figure S4). These interactions were all negative, indicating that the within-pair effect of clinical autism on brain connectivity between these regions decreased with increasing influence of age. Despite a larger amount of trait discordant compared to diagnosis discordant twin pairs, we did not find any evidence for a modulating effect of age on the within-pair association between autistic traits and connectivity. The interaction effects between age and autism diagnosis should be interpreted with caution, due to the limited number of twin pairs discordant for clinical autism. Still—if validated in further studies—this is a clinically interesting observation in that it might reflect compensatory mechanisms affecting structural network organization, potentially taking place during adolescence and early adulthood.

### Sex differences

We conducted follow-up analyses by splitting the sample with regard to biological sex assigned at birth. While the main results did not differ significantly between males and females, the association between autistic traits and connectivity between left hippocampus and left parahippocampal gyrus survived the correction only in females. This association might hence be more strongly driven by females and consequently be specific to samples including more females than the average autism study. When looking for sex differences within all included connections, we found that the within-pair association between autistic traits and connectivity between the brainstem and the right inferior frontal gyrus differed statistically significantly between males and females. Studies investigating sex differences in neural correlates of autism are still scarce today, mostly focusing on brain morphometry measures (GM and WM volumes or cortical thickness) or intrinsic functional connectivity^8^. These studies indicate that clinical autism is associated with partly different brain regional and intrinsic functional connectivity alterations in males and females, especially involving regions that also show sex differences in the general population^8^. Further, twin studies from our group assessing sex specific brain morphological correlates of clinical autism and autistic traits in twin samples that partly overlap with the sample of the present study found that within-pair differences in overall autistic traits and repetitive behaviors and restricted interests were associated with more brain-morphological alterations in females, while social cognition abilities were associated with more brain alterations in males^24–26^. Together, these studies suggest that autistic trait differences between twins, which are likely to be influenced by non-shared environmental factors, are differentially linked to brain structural alterations in males and females.

Here, we found the connectivity between brainstem and the right inferior frontal gyrus to be negatively associated with autistic traits in male, but positively in female twins compared to their co-twins. We already discussed the potential role of the brainstem in autism with regard to our main results. The right inferior gyrus plays a crucial role in detecting relevant cues^52^ and its interaction with the brainstem has been suggested to be relevant during tasks where attentional demands fluctuate in an unpredictable manner^53^. Increased connectivity in females and reduced in males with higher autistic traits compared to their co-twins might hence reflect more autistic traits-related difficulties in handling unpredictable attentional demands in males and compensatory processes specific to females.

## Strength and limitations

Recruiting primarily twin pairs with marked differences in autistic traits made this study more sensitive for detecting within-pair associations with autistic traits, but also prevented us from conducting classical twin modeling of the quantitative genetic and shared vs non-shared environmental effects since these estimates would have been biased.

Power analysis for our twin analysis is not straight forward, due to the non-independence of observations and due to our recruitment strategy, however, we aimed to approximate the questions using G*Power (version 3.1.9.2), assuming n = 77 pairs differing on the SRS-2. While the sample is comparatively large for a neuroimaging twin study on autism, our power calculation indicated a power of 85.6% in order to detect medium sized effects (β > .3) at α = 0.05. A much larger sample (n = 714 discordant twin pairs) would have been required to detect small effects (β = .1) at a power of 80%. Further, the FDR-correction for the relatively large number of 859 tested connections might have increased the likelihood of type-II errors. Therefore, we included uncorrected Z-maps in the supplementary material in order to provide an overview over sub-threshold effects. These revealed visually relatively similar patterns of both increased and decreased connectivity in association with clinical autism and autistic traits, indicating that rather than differing fundamentally in their effects on the overall connectivity pattern, clinical autism and autistic traits might only differ with respect to the most robust associations with structural connectivity.

Our study had a wide age range and an even distribution of males and females, increasing the generalizability across ages and sexes, but this variability might on the other hand have prevented us from detecting sub-group specific effects. Further, the age distribution was not even across the sample, with fewer individuals especially in the oldest age categories, which is another reason to treat the age-interaction effects with caution. Moreover, age-interaction effects are not necessarily linear. Since we are lacking the power to compare different models to find the best fitting shape, the linear regressor serves as the simplest model, which will likely capture trends even if they are not actually linear. In general, the conclusions that can be drawn from cross-sectional studies with regard to development are limited. Longitudinal studies, ideally assessing individuals with and without autism from a younger age, are required in order to differentiate causal from compensatory mechanisms.

Excluding individuals with an IQ < 75 and individuals with insufficient brain imaging data quality has likely reduced the noise in the data, but restricted the sample to individuals in the normal IQ-range who could lie relatively still in the scanner. A comparison between the excluded and included sample (see Supplementary Text “Supplementary results” section and Supplementary Table S2) revealed that the excluded sample was not only characterized by a lower IQ, but it was also younger on average, and included a greater proportion of males and individuals with clinical autism, ADHD, and other NDDs. It might be expected that younger individuals with NDD diagnoses and lower IQ would have more difficulties to keep still in an MRI scanner, potentially leading to DTI data quality issues. The differences between included and excluded sample indicate that our clinical sub-cohort might not be representative for autistic/NDD populations, which limits the generalizability of our findings. In addition, more than twice as many autistic males than females had to be excluded, which might explain the unusually balanced sex distribution in the included sample, together with a strategy to include both sexes and the diagnostic label being based on our own assessment rather than health care providers. Hence, our results reflect changes in connectivity associated with autism and autistic traits in a sample that contains more females and individuals with higher average IQ scores, and fewer comorbidities than what might typically characterize an autistic cohort.

In this study, we were unable to differentiate effects of biological sex and gender identity, since no information on gender identity was collected. Future studies should take both aspects into account.

Finally, twin cohorts differ from non-twin cohorts in several ways. For instance, twins are more frequently born prematurely and suffer more often from growth restrictions^54^. Hence, we cannot exclude the possibility that they also differ from non-twin samples in terms of brain connectivity.

## Conclusions

Using a data-driven approach and a within-twin pair design, we found evidence for reduced brainstem-occipital connectivity in association with clinical autism, and reduced connectivity of the hippocampus to parahippocampal and fusiform gyri in association with autistic traits, beyond familial confounding and across sexes and ages. These associations were significant in DZ twins alone and attenuated in MZ twins despite pointing in the same direction, potentially indicating both genetic and environmental contributions. The negative interaction effects between clinical autism and age on brain connectivity might reflect compensatory processes. We found also evidence for sex differences in a within-pair association between autistic traits and structural connectivity, in line with former evidence for a qualitatively different neurobiology of autism in females and males. Overall, our within-pair results reflect structural brain correlates of clinical autism and autistic traits that are specific to individuals within a normal IQ range, to a larger extent influenced by non-shared environmental factors compared to case–control studies, and less specific to the male expression of the autism phenotype as compared to the majority of previous studies on structural connectivity in autism.

### Supplementary Information

Supplementary Information.

## Data Availability

The datasets analyzed during the current study are not publicly available due to data protection reasons, but are available from the corresponding author on reasonable request. Access will be given to named individuals in accordance with ethical procedures governing the reuse of sensitive data, including completion of a formal data sharing agreement.

## Abbreviations

DZ: Dizygotic
FA: Fractional anisotropy
FFA: Fusiform face area
FOV: Field of view
IQ: Intelligence quotient
MRI: Magnetic resonance imaging
MZ: Monozygotic
NDD: Neurodevelopmental disorder
NT: Neurotypical
TR: Repetition time
WM: White matter
