# Supplementary material for: Reduced brain connectivity along the autism spectrum controlled for familial confounding by co-twin design
Source: Sci Rep. 2023 Aug 12;13:13124. doi: 10.1038/s41598-023-39876-y (PMC10423238; doi:10.1038/s41598-023-39876-y)
Supplement: Supplementary file 1 — Supplementary Information. [file 41598_2023_39876_MOESM1_ESM.pdf]

SUPPLEMENTARY MATERIAL

Reduced Brain Connectivity Along the Autism Spectrum Controlled for Familial Confounding by Co-Twin Design

Neufeld, Janina, PhD[1*], Maier, Simon, PhD[2], Revers, Mirian, MD[1], Reisert, Marco, PhD[2], Kuja-Halkola, Ralf, PhD[3], Tebartz van Elst, Ludger, Prof.[2], & Bölte, Sven, Prof.[1,4,5]

[1]Center of Neurodevelopmental Disorders (KIND), Centre for Psychiatry Research; Department of Women's and Children's Health, Karolinska Institutet & Stockholm Health Care Services, Region Stockholm, Stockholm, Sweden.

[2]Section for Experimental Neuropsychiatry, Department for Psychiatry & Psychotherapy, Medical Center University of Freiburg, Freiburg, Germany

[3]Department of Medical Epidemiology and Biostatistics, Karolinska Institutet, Stockholm, Sweden

[4]Child and Adolescent Psychiatry, Stockholm Health Care Services, Region Stockholm, Stockholm, Sweden.

[5]Curtin Autism Research Group, Curtin School of Allied Health, Curtin University, Perth, Western Australia.

*corresponding author

<u>**Table of contents**</u>

1. Exclusion procedure…………………………………..……………………………....3

2. Supplementary results……………………………………………………………….....5

Supplementary Table S1: The 56 LPBA40 atlas regions…………………………………….8

Supplementary Table S2: Comparison of included and excluded sample……….………..9

Supplementary Table S3: Covariate effects….…………………………………….……...11

Supplementary Table S4: Interactions between clinical autism and age within-pairs..…….12

Supplementary Fig. S1: Z-map of all included connections in association with clinical autism…………………………………………………………………………………....13

Supplementary Fig. S2: Z-map of all included connections in association with autistic traits...…………………………………………………………………………………………14

Supplementary Fig. S4: Connections showing significant age by diagnosis interactions with age…………………………………………………………………………………………….15

Supplementary Fig. S5: Z-map of the interaction between clinical autism and age on structural connectivity………………………………………………………………………………...16

Supplementary Fig. S6: Z-map of the interaction between autistic traits and age on structural connectivity.……………………………………………………………….................17

Supplementary Fig. S7: Visualization of the age by autism diagnosis interaction effect on connectivity…………………………………………………………………………………...18

Supplementary Fig. S8: Visualization of the sex interaction effect on the association between connectivity and autistic traits……………………………………………………………………18

1. Exclusion procedure

From the entire RATSS sample collected until September 2019 (N=420), the first 42 pairs (N=84) were excluded because their signal-to-noise ratio of the diffusion data was considerably lower than in the rest of the sample due to a necessary scanner update after they had been acquired. The lower signal to noise ratio was the most common exclusion reason, followed by exclusion due to insufficient quality diffusion data (e.g. due to severe movement-related artifacts, 60 participants) and exclusion because the co-twin had to be excluded (55 participants). Further, we excluded 20 individuals were no DTI data had been acquired or the data could not be processed, 11 individuals with an IQ below 75, five individuals where no SRS-2 data were acquired, six individuals from opposite sex pairs, and five individuals because more than one twin pair per family had been assessed.

2. Supplementary results

2.1 Differences between included and excluded sample

In order to investigate potential differences between the included and excluded sample assessed within RATSS, we performed Chi^2 tests to test for differences in sex or zygosity and in the numbers of participants diagnosed with clinical autism, ADHD or other NDDs. Note that categorical variables overlapped, so that individuals with multiple diagnoses contributed to the effects of each of those diagnoses. Further we performed t-tests in order to compare the included and excluded sample in categorical variables, namely age, autistic traits (total SRS-2 and sub-scale for autism mannerisms, indicating repetitive behaviors / restricted interests) and IQ.

The excluded sample differed significantly from the included sample in several variables (see **Supplementary Table 2**). More specifically, the excluded sample contained more males and more individuals with clinical autism, ADHD and other NDDs. Interestingly, more than twice as many autistic males (n=48) than females (n=23) had to be excluded, while sex was rather balanced in the included sample, both for autistic and non-autistic individuals. Further, the excluded sample was overall younger, had higher mean autistic traits and a lower mean IQ. The latter was expected, given that we explicitly excluded individuals with an IQ below 75. Further, younger participants and participants with lower IQ and NDDs, respectively, are likely to have more difficulties to complete the MRI data acquisition while keeping still.

There was no statistically significant difference between included and excluded sample in terms of zygosity and proportion of individuals with non-NDD psychiatric diagnoses (mainly depression and anxiety).

2.2 Group differences in autistic traits, age and IQ

For descriptive analysis, we divided the sample into individuals diagnosed with clinical autism (n = 24), NT individuals (n = 90) and individuals diagnosed with other NDD or psychiatric

conditions but without clinical autism (n = 60). Individuals diagnosed with clinical autism had on average more autistic traits compared to both NT individuals (*t(29.23) = 10.48, p =* $2.1*10^{-11}$) and individuals with other NDD or psychiatric diagnoses (*t(36.29) = 8.29, p =* $6.7*10^{-10}$). Further, individuals with other NDD or psychiatric diagnoses had on average higher autistic traits than NT individuals (*t(111.44) = 2.79, p = .006*). The proportion of females was 54% in the clinical autism sample, 54% in the NT sample, and 63% in individuals with other NDD or psychiatric diagnoses. The gender distribution did not differ significantly between these three groups (*clinical autism vs NT:* $Chi^2(1)$ *= .27, p = .60; clinical autism vs other diagnoses:* $Chi^2(1)$ *= 1.50, p = .22; other diagnoses vs NT:* $Chi^2(1)$ *= .83, p = .36*). There were no significant group differences in age (*clinical autism vs NT: t(39.21) = -1.88, p-value = .068; clinical autism vs other diagnoses: t(49.72) = -1.67, p = .10; other diagnoses vs NT: t(120.49) = -.08, p = .94*) or IQ (*clinical autism vs NT: t(29.90) = -.29, p = .77; clinical autism vs other diagnoses: t(32.91) = -.50, p = .62; other diagnoses vs NT: t(128.38) = -1.46, p = .15*).

2.3 Within-pair differences in IQ

The mean IQ differences between twins were moderate, both in the whole sample (*mean = 9.83; SD = 8.39*) and in the 16 pairs discordant for clinical autism (*mean = 14.19, SD = 11.86).* Within-pair differences of autism-discordant pairs and twin pairs where no individual was diagnosed with clinical autism did not differ significantly *(t(17.98)= 1.05, p-value = .31*).

2.4 Within-twin pair effects of covariates on structural connectivity

None of the covariates were significantly associated with any of the connections that were found to be associated with clinical autism or autistic traits after FDR-correction. Taking all 859 connections into account, a few associations were significant and these were almost identical for the model with autistic traits and the model with clinical autism as main predictors. Please see **Supplementary Table S3** for the statistics derived from the model with autistic traits as

main predictor. Having an NDD diagnosis other than autism was associated with reduced connectivity of the left precentral gyrus with both left supramarginal and left superior temporal gyrus, as well as reduced connectivity between right cingulate and right angular gyrus within pairs. Having a non-NDD psychiatric diagnosis and having a higher IQ were associated with increased connectivity between left caudate and left inferior frontal gyrus. Having a higher IQ compared to one's twin was further associated with reduced connectivity between left gyrus rectus and left middle orbitofrontal gyrus.

2.5 Z-maps of all assessed associations (uncorrected)

The uncorrected Z-maps of the associations between connectivity as the outcome predicted by clinical autism (**Supplementary Fig. S2**) and autistic traits (**Supplementary Fig. S3**), respectively, revealed overall relatively similar patterns of increases and decreases in connectivity. Comparing the two Z-maps visually, our impression is that associations with clinical autism and autistic traits as predictors differed primarily in respect to which Z-values were most extreme and which associations survived the corrections for multiple comparisons, rather than leading to fundamentally different patterns of altered connectivity. Associations surviving the correction for one measure had also relatively high Z-values in the same direction for the other, albeit below the significance threshold. However, negative Z-values reached more extreme values for clinical autism compared to autistic traits.

Further, it is apparent that both clinical autism and autistic traits were also associated with extended patterns of (non-significant) increases in connectivity within twin-pairs rather than uniformly being associated with reduced connectivity – especially of occipital and occipito-temporal brain regions to temporal and parietal regions and of brain stem to frontal brain regions.

2.6 Across cohort associations of clinical autism and autistic traits with brain connectivity

In order to complement our within-pair analysis, we also performed linear regressions across the cohort using the same statistical framework, treating twins as individuals but adjusting standard errors for twin clustering. The latter model is more similar to regression analyses in non-twin samples and hence more comparable to the majority of previous studies assessing relationships between clinical autism / autistic traits and WM connectivity, but these results do not directly reflect associations in the general population (due to the sampling bias). Across the cohort, we adjusted additionally for sex and age (these variables are implicitly controlled for in the within-pair analysis). None of the associations with clinical autism or autistic traits that were found to be significant within twin pairs in the main analysis survived the correction for multiple comparisons across the cohort.

However, other associations were found. More specifically, clinical autism was associated with reduced connectivity (lower stream line count) of the left inferior frontal gyrus with the left caudate nucleus (*-.674 (-1.001, -.347), SE = .167, corrected p = .045, uncorrected* $p = 5.3*10^{-5}$) and with the left superior parietal gyrus, respectively (*b (95% CI) = -.777 (-1.069, -.485), SE = .149, corrected* $p = 1.5*10^{-4}$, *uncorrected* $p = 1.8*10^{-7}$). Clinical autism was additionally negatively associated with connectivity between the right postcentral gyrus and the right lateral orbitofrontal gyrus (*b (95% CI) = -.612 (-.873, -.351), SE = .133, corrected p = .004, uncorrected* $p = 4.4*10^{-6}$). No associations with autistic traits and brain connectivity across the cohort survived the correction.

Since these associations were only found across the cohort, we suggest that these connectivity alterations are influenced by familial factors, such as genetics or upbringing. This means that co-twins of twins with clinical autism who themselves do not fulfil diagnostic criteria might nevertheless share the connectivity alterations between these regions to some extent.

Supplementary Tables

Supplementary Table S1: The 56 LPBA40 atlas regions

numeric label	Name label	numeric label	Name label
21	L superior frontal gyrus	65	L inferior occipital gyrus
22	R superior frontal gyrus	66	R inferior occipital gyrus
23	L middle frontal gyrus	67	L cuneus
24	R middle frontal gyrus	68	R cuneus
25	L inferior frontal gyrus	81	L superior temporal gyrus
26	R inferior frontal gyrus	82	R superior temporal gyrus
27	L precentral gyrus	83	L middle temporal gyrus
28	R precentral gyrus	84	R middle temporal gyrus
29	L middle orbitofrontal gyrus	85	L inferior temporal gyrus
30	R middle frontal gyrus	86	R inferior temporal gyrus
31	L lateral orbitofrontal gyrus	87	L parahippocampal gyrus
32	R lateral orbitofrontal gyrus	88	R parahippocampal gyrus
33	L gyrus rectus	89	L lingual gyrus
34	R gyrus rectus	90	R lingual gyrus
41	L postcentral gyrus	91	L fusiform gyrus
42	R postcentral gyrus	92	R fusiform gyrus
43	L superior parietal gyrus	101	L insular cortex
44	R superior parietal gyrus	102	R insular cortex
45	L supramarginal gyrus	121	L cingulate gyrus
46	R supramarginal gyrus	122	R cingulate gyrus
47	L angular gyrus	161	L caudate
48	R angular grus	162	R caudate
49	L precuneus	163	L putamen
50	R precuneus	164	R putamen
61	L superior occipital gyrus	165	L hippocampus
62	R superior occipital gyrus	166	R hippocampus
63	L middle occipital gyrus	~~181~~	~~cerebellum~~
64	R middle occipital gyrus	182	brainstem

Note. *R = right and L = left hemisphere; we excluded the cerebellum (region 181) from our analysis due to incomplete coverage in many participants.*

Supplementary Table S2: Comparison between included and excluded sample

	**Included sample (n=174)**	**Excluded sample (n=246)**	**statistics**
**Female / male sex**	98 / 76	97 / 149	**p (Chi^2) < .001**
**MZ / DZ (undetermined)**	96 / 78 / 0	128 / 110 (8)	p (Chi^2) = .86
**Clinical autism %**	14 %	29 %	**p (Chi^2) < .001**
**ADHD %**	18 %	34 %	**p (Chi^2) < .001**
**Other NDD (not autism / ADHD) %**	10 %	26 %	**p (Chi^2) < .01**
**Non-NDD psychiatric diagnosis %**	29 %	23 %	p (Chi^2) = .16
**Age range years**	8-36	8-28	-
**Mean age (SD)**	19.51 (6.50)	14.65 (4.79)	**p (t) < .001**
**Mean SRS-2 (SD)**	32.38 (28.04)	48.67 (36.05)	**p (t) < .001**
**Mean RRBI (SD)**	4.57 (6.25)	7.62 (8.00)	**p (t) < .001**
**Mean IQ (SD)**	103.27 (14.08)	96.67 (16.48)	**p (t) < .001**

Note. *MZ = monozygotic; DZ = dizygotic; undetermined = zygosity could not be determined since genetic data were not available and the questionnaire data was inconclusive; Other NDD = neurodevelopmental diagnoses other than autism or ADHD; SRS-2 = social responsiveness scale second version; RRBI = repetitive behaviors and restricted interests assessed with the autism mannerism sub-scale of the SRS-2; IQ = general intellectual ability score from the Wechsler Intelligence scale for adults or children.*

Supplementary Table S3: Covariate effects

**Anatomical regions**	***b* (95% CI)**	***SE***	***p* corr.**	***p* uncorr.**
Other NDD diagnoses				
**L supramarginal g. – L precentral g.**	*-.809 (-1.195, -.422)*	*.197*	*.035*	$4.1*10^{-5}$
**L sup. temporal g. - L precentral g.**	*-.945 (-1.293, -.598)*	*.177*	$8.3*10^{-5}$	$9.7*10^{-8}$
**R cingulate g. – R angular g.**	*-1.592 (-2.207, -.977)*	*.314*	$3.4*10^{-4}$	$4.0*10^{-7}$
psychiatric non-NDD diagnoses				
**L caudate – L inf. frontal g.**	*.628 (.341, .915)*	*.147*	*.016*	$1.8*10^{-5}$
IQ				
**L g. rectus – L mid. orbitofrontal g.**	*-.040 (-.057, -.022)*	*.009*	*.010*	$1.2*10^{-}$
**L caudate – L inf. frontal g.**	*.032 (.018, .047)*	*.007*	*.008*	$9.0*10^{-6}$

Note. *b = regression coefficient, 95%CI = 95% confidence interval of the regression coefficient, SE = standard error, p corr. = FDR-corrected p-value, p uncorr. = uncorrected p-value, L = left, R = right, g. = gyrus, sup. = superior, mid. = middle, inf. = inferior. These statistics were derived from the models with autistic traits as the main predictor, but were very similar compared to the models with clinical autism as main predictor.*

Supplementary Table S4: Interactions between clinical autism and age within-pairs

Anatomical regions	*b (95% CI)*	*SE*	*p corr.*	*p uncorr.*
**L sup. occipital g. – L precentral g.**	*-.109 (-.152, -.065)*	*.022*	*.001*	$9.6*10^{-7}$
**L mid. occipital g. – L precentral g.**	*-.108 (-.146, -.069)*	*.020*	$4.3*10^{-5}$	$4.8*10^{-8}$
**L inf. occipital g. – L postcentral g.**	*-.152 (-.209, -.095)*	*.029*	$1.6*10^{-4}$	$1.8*10^{-7}$
**L inf. occipital g. – L precentral g.**	*-.173 (-.226 -.119)*	*.027*	$3.4*10^{-7}$	$2.6*10^{-10}$
**L inf. occipital g. – L inf. front. g.**	*-.148 (-.220, -.076)*	*.037*	*.047*	$5.2*10^{-5}$
**R inf. occipital g. – R mid. front. g.**	*-.184 (-.242, -.126)*	*.030*	$5.1*10^{-7}$	$5.7*10^{-10}$
**R inf. occipital g. – R sup. front. g**	*-.143 (-.206, -.081)*	*.032*	*.007*	$7.9*10^{-6}$
**R cuneus – R mid. front. g.**	*-.111 (-.165, -.057)*	*.027*	*.049*	$5.5*10^{-5}$
**R cuneus – R sup. front. g.**	*-.111 (-.160, -.063)*	*.025*	*.005*	$5.9*10^{-6}$
**L lingual g. – L mid. front. g.**	*-.185 (-.274, -.095)*	*.046*	*.047*	$5.3*10^{-5}$
**R lingual g. – R angular g.**	*-.118 (-.164, -.073)*	*.023*	$3.5*10^{-4}$	$3.9*10^{-7}$
**R lingual g. – R mid. front. g.**	*-.126 (-.183, -.069)*	*.029*	*.015*	$1.6*10^{-5}$
**L fusiform g. – L inf. front. g.**	*-.100 (-.135, -.056)*	*.020*	*.002*	$2.4*10^{-6}$
**L fusiform g. – L mid. front. g.**	*-.101 (-.150, -.052)*	*.025*	*.044*	$4.9*10^{-5}$
**R putamen – R angular g.**	*-.080 (-.117, -.044)*	*.018*	*.011*	$1.2*10^{-5}$

Note. *b = regression coefficient, 95%CI = 95% confidence interval of the regression coefficient, SE = standard error, p corr. = FDR-corrected p-value, p uncorr. = uncorrected p-value, L = left, R = right, g. = gyrus, sup. = superior, mid. = middle, inf. = inferior, front. = frontal. All estimates (except one) were negative, indicating weaker within-pair associations in older twin pairs.*

Supplementary Figures

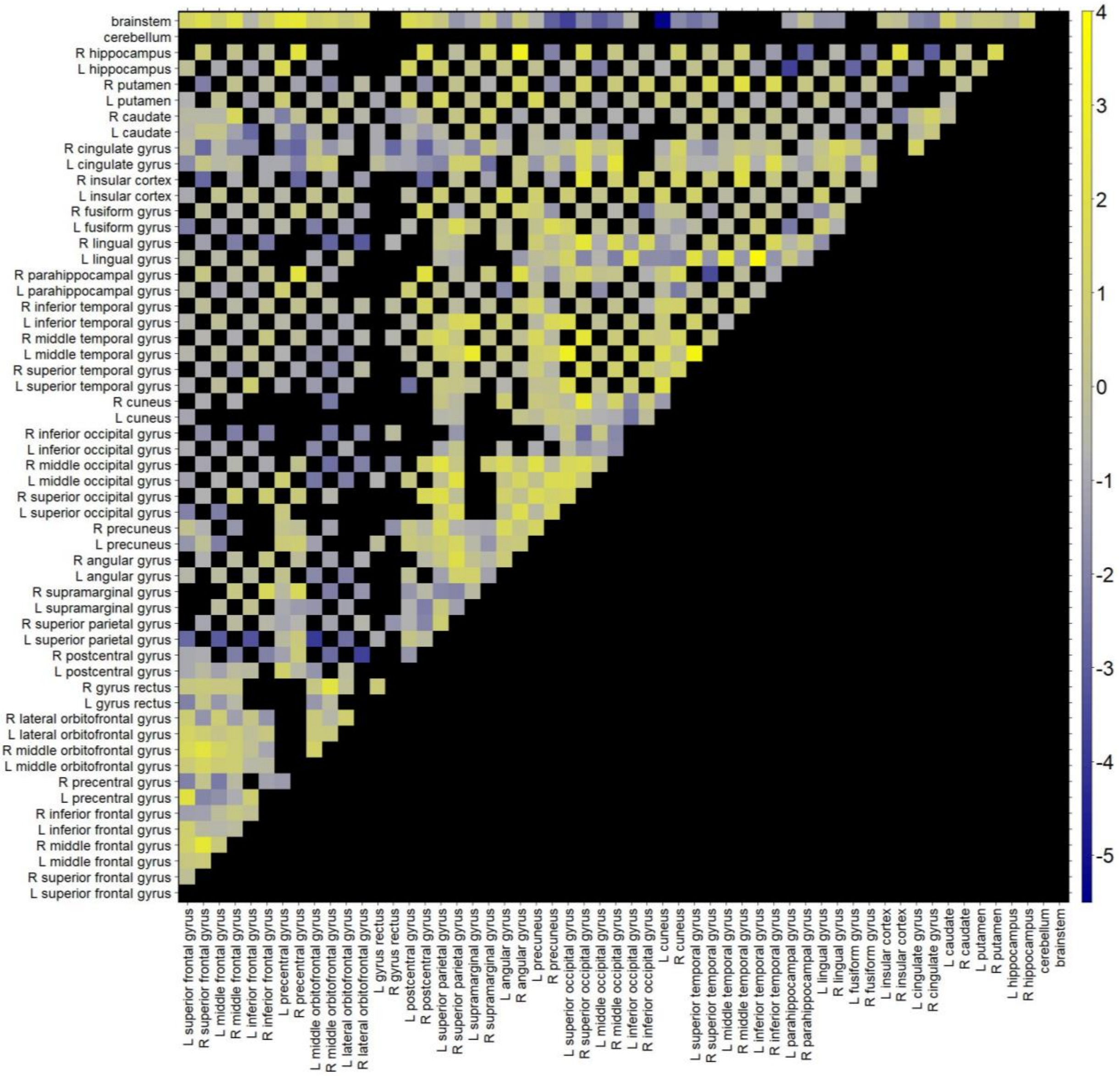

Fig. S2: Z-map of all 859 included connections in association with clinical autism. *The legend shows the color range of Z-values (-5.5 to +4) and yellow squares depict positive and blue squares negative within-pair associations between clinical autism and connectivity while adjusting for IQ and other neurodevelopmental and psychiatric diagnosis. Black squares represent excluded connections.*

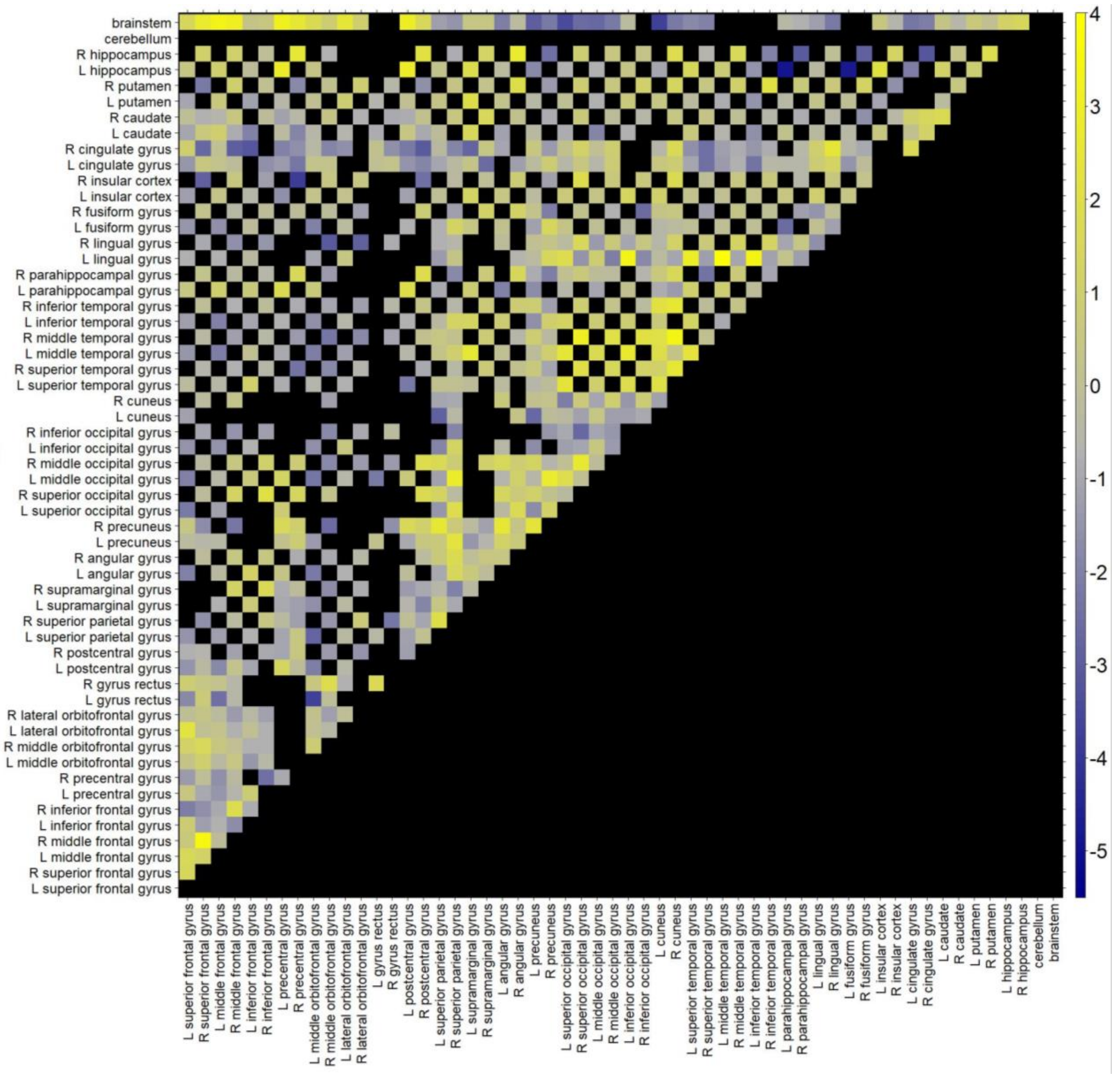

***Fig. S3**: Z-map of all 859 included connections in association with autistic traits.* *The legend shows the color range of Z-values (-5.5 to +4) and yellow squares depict positive and blue squares negative within-pair associations between autistic traits and connectivity while adjusting for IQ and other neurodevelopmental and psychiatric diagnosis. Black squares represent excluded connections.*

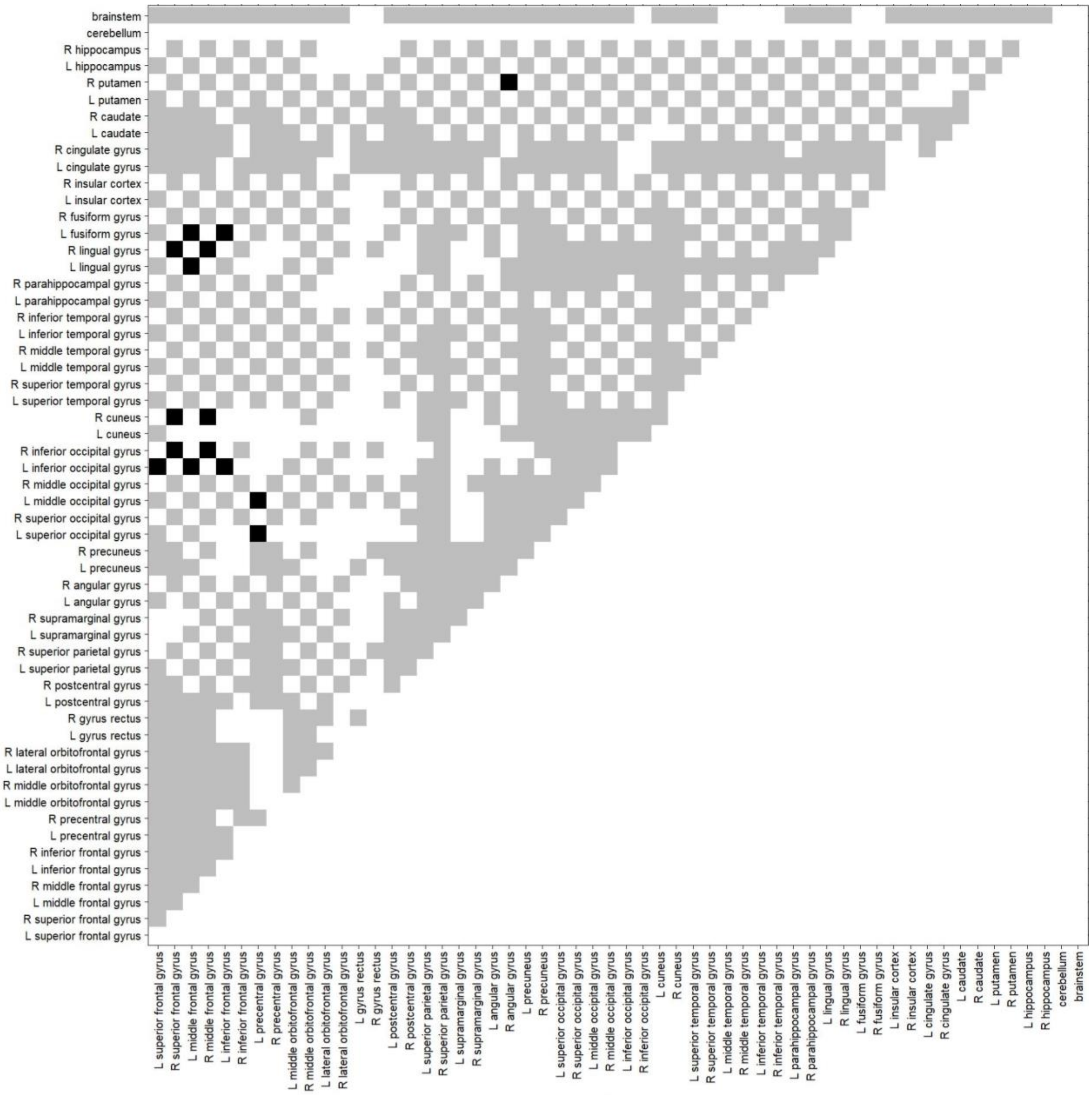

***Fig. S4**: **Connections showing significant age by clinical autism diagnosis interactions**. 15 connections showed a significant (negative) clinical autism diagnosis by age interaction within pairs, marked in black, included connections are marked in grey. White squares represent excluded connections.*

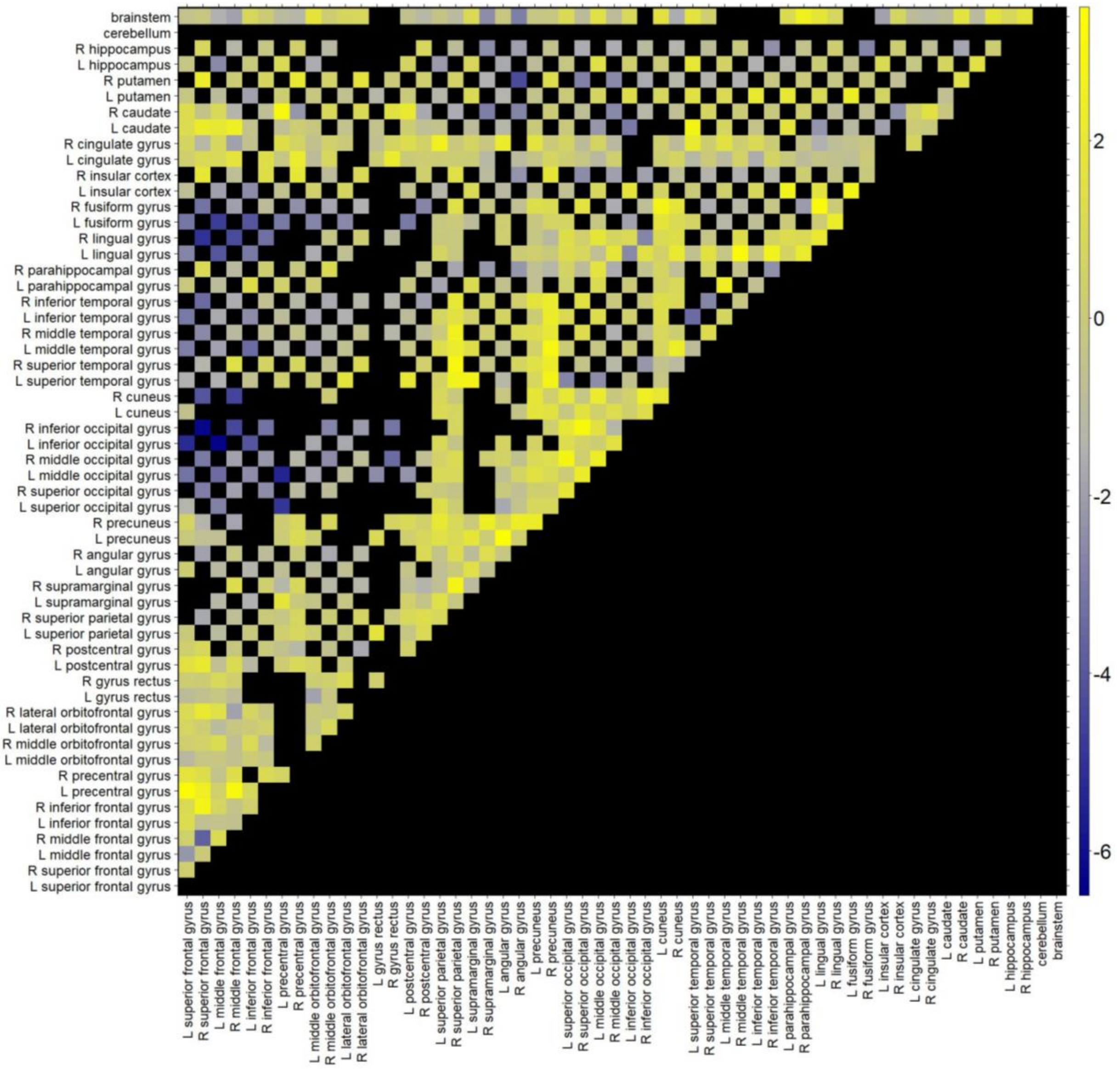

Fig S5: Z-map of the interaction between clinical autism and age on structural connectivity*. Z-values ranged from -6.3 to 2.8 and yellow squares depict positive and blue squares negative within-pair age interaction effects. Black squares represent excluded connections.*

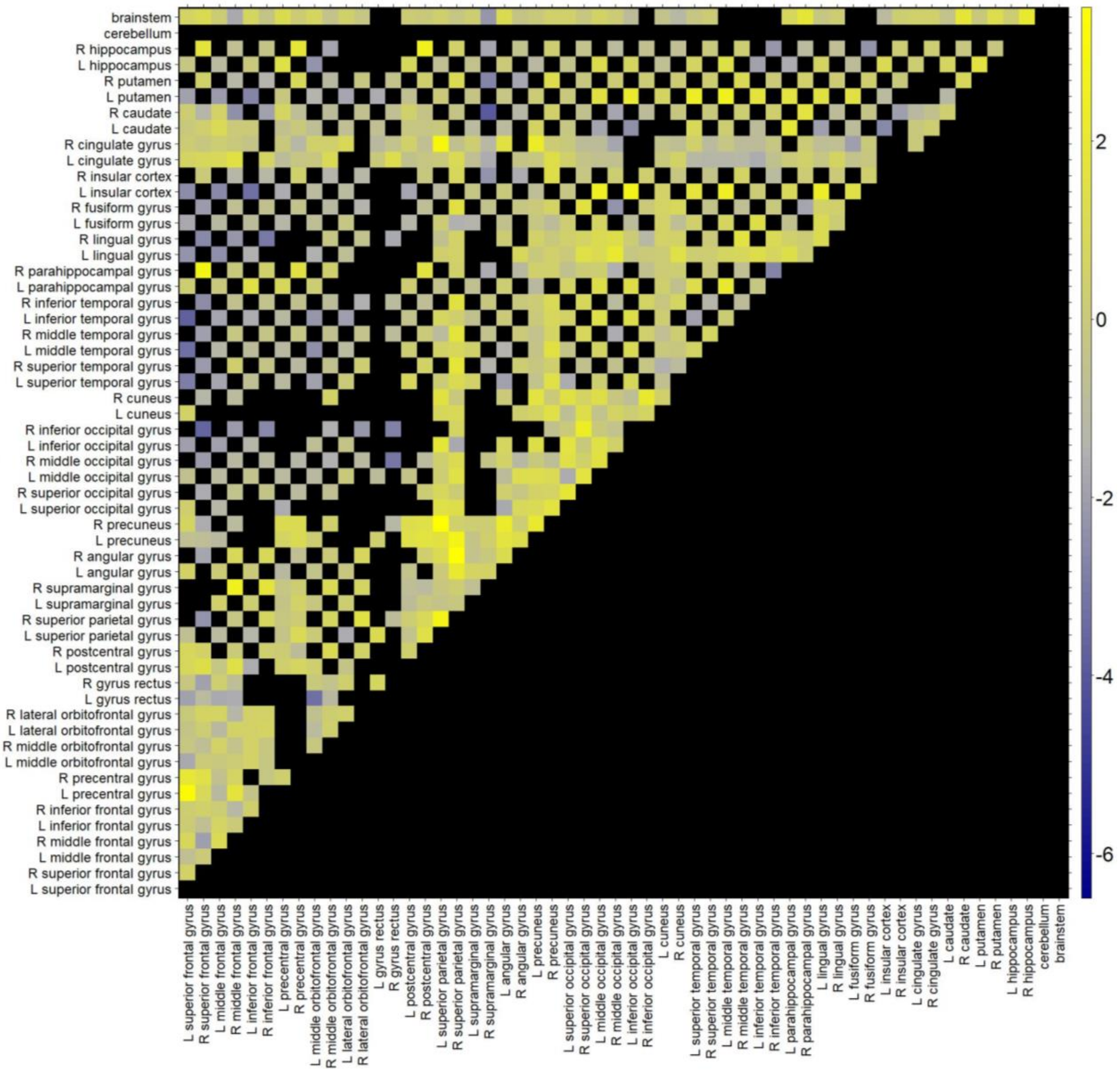

***Fig S6: Z-map the interaction between autistic traits and age on structural connectivity**. Z-values ranged from -4 to 3.5 and yellow squares depict positive and blue squares negative within-pair age interaction effects. The legend was set to range from -6.5 to 3.5 in order in order to be better able to compare the patterns for age-interactions with clinical autism and autistic traits. Black squares represent excluded connections.*

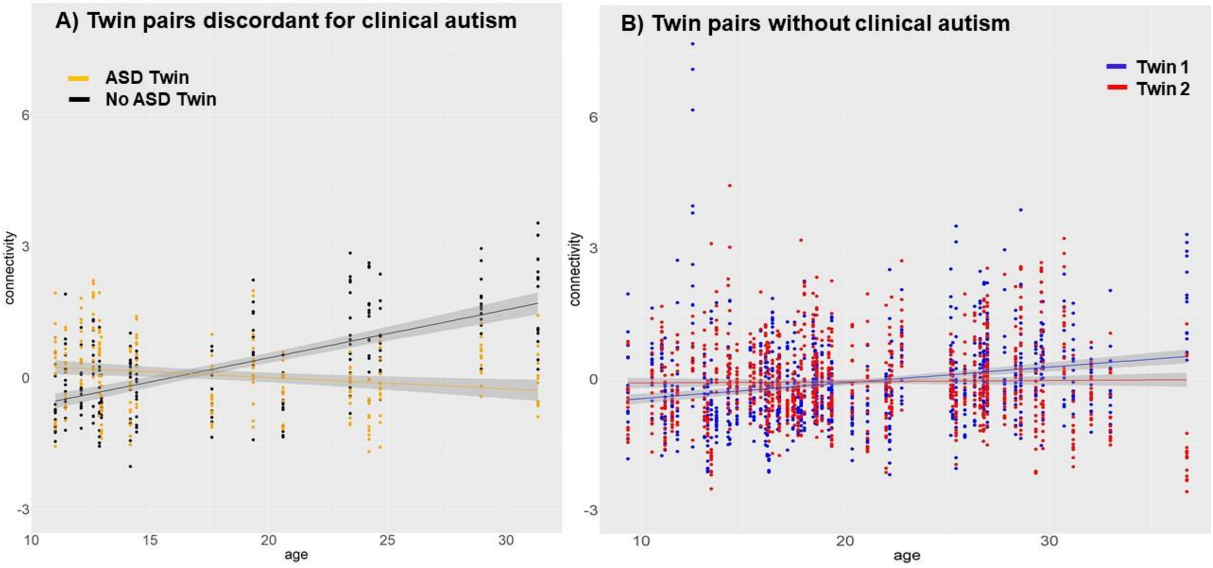

Fig S7: Visualization of the age by autism diagnosis interaction effect on connectivity. *The interaction between clinical autism and age was significant for 15 connections. For these, we extracted the standardized connectivity values for A) autistic twins (orange) and their non-autistic co-twins (black) and B) for twin pairs where no twin had clinical autism (twins are labelled twin 1 and twin 2 depending on which twin was labelled twin 1 and 2 at recruitment. The plot does not represent the model results, but approximates visualizing them in a simpler fashion. What we can see that connectivity estimates of the connections showing age-interaction effects tend to be on average higher in older compared to younger individuals without clinical autism, especially in co-twins of autistic twins, while there seems to be a tendency of these connections to be weaker in older compared to younger individuals with autism.*

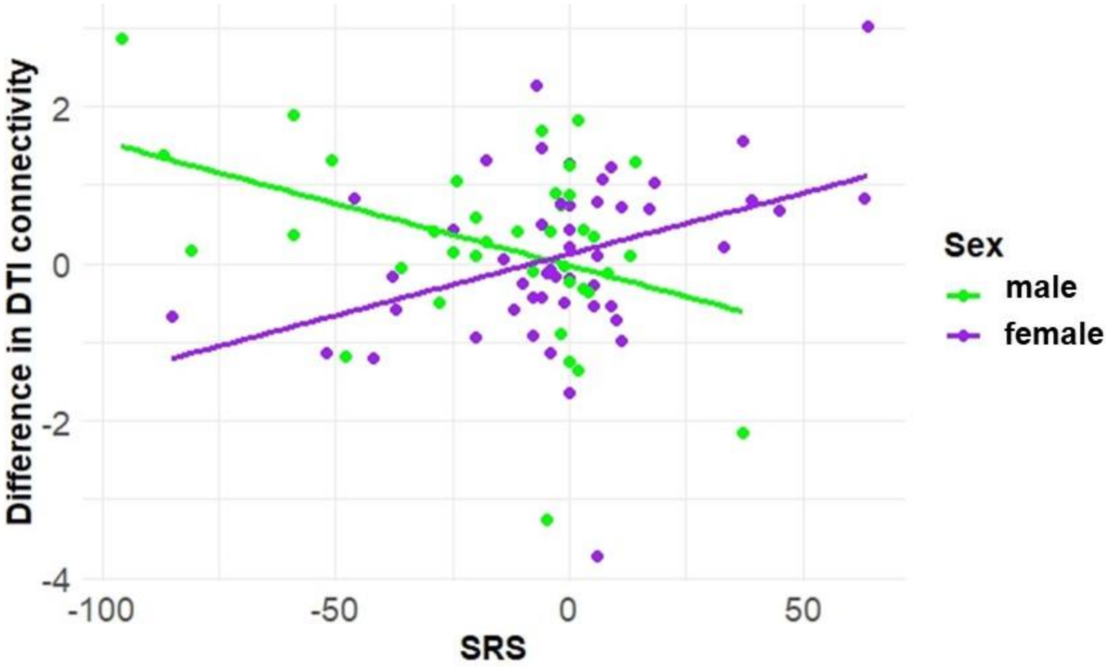

Fig S8: Visualization of the sex interaction effect on the association between connectivity and autistic traits. *For one connection, between the brainstem and the right inferior frontal gyrus, the within pair association between connectivity and SRS-2 differed between females and males. The plot does not represent the model results, but approximates visualizing them in a simpler fashion, using the within-pair differences in structural connectivity as function of the within-pair differences in autistic traits.*
